# Lactic acid from vaginal microbiota enhances cervicovaginal epithelial barrier integrity by promoting tight junction protein expression

**DOI:** 10.1186/s40168-022-01337-5

**Published:** 2022-08-31

**Authors:** David Jose Delgado-Diaz, Brianna Jesaveluk, Joshua A. Hayward, David Tyssen, Arghavan Alisoltani, Matthys Potgieter, Liam Bell, Elizabeth Ross, Arash Iranzadeh, Imane Allali, Smritee Dabee, Shaun Barnabas, Hoyam Gamieldien, Jonathan M. Blackburn, Nicola Mulder, Steven B. Smith, Vonetta L. Edwards, Adam D. Burgener, Linda-Gail Bekker, Jacques Ravel, Jo-Ann S. Passmore, Lindi Masson, Anna C. Hearps, Gilda Tachedjian

**Affiliations:** 1 Life Sciences Discipline, Burnet Institute, 85 Commercial Road, Melbourne, VIC 3004 Australia; 2 Department of Microbiology, Monash University, Clayton, VIC 3168 Australia; 3 Division of Medical Virology, Department of Pathology, University of Cape Town, Cape Town, 7925 South Africa; 4 Division of Biomedical Sciences, University of California Riverside School of Medicine, Riverside, CA 92521 USA; 5 Computational Biology Division, Department of Integrative Biomedical Sciences, University of Cape Town, Cape Town, 7925 South Africa; 6 Division of Chemical and Systems Biology, Department of Integrative Biomedical Sciences, University of Cape Town, Cape Town, 7925 South Africa; 7 Centre for Proteomic and Genomic Research, Cape Town, 7925 South Africa; 8 Laboratory of Human Pathologies Biology, Department of Biology, Faculty of Sciences, Mohammed V University in Rabat, 1014 Rabat, Morocco; 9 Center for Global Infectious Disease Research, Seattle Children’s Research Institute, Seattle, WA 98101 USA; 10 Family Centre for Research with Ubuntu, Stellenbosch University, Cape Town, 7505 South Africa; 11 Institute of Infectious Disease and Molecular Medicine (IDM), University of Cape Town, Cape Town, 7925 South Africa; 12 Centre for Infectious Diseases Research (CIDRI) in Africa Wellcome Trust Centre, University of Cape Town, Cape Town, 7925 South Africa; 13 Institute for Genome Sciences, University of Maryland School of Medicine, Baltimore, MD 21201 USA; 14 Department of Microbiology and Immunology, University of Maryland School of Medicine, Baltimore, MD 21201 USA; 15 Center for Global Health and Diseases, Case Western Reserve University, Cleveland, OH 44106 USA; 16 Department of Obstetrics and Gynecology, University of Manitoba, Winnipeg, Canada; 17 Department of Medicine Solna, Karolinska Institutet, Stockholm, Sweden; 18 Desmond Tutu HIV Centre, University of Cape Town, Cape Town, 7925 South Africa; 19 Centre for the AIDS Programme of Research in South Africa, Durban, 4013 South Africa; 20 National Health Laboratory Service, Cape Town, 7925 South Africa; 21 Central Clinical School, Monash University, Melbourne, 3004 Australia; 22 Department of Microbiology and Immunology at the Peter Doherty Institute for Infection and Immunity, University of Melbourne, Melbourne, VIC 3010 Australia

**Keywords:** Female reproductive tract, Lactic acid, Vaginal microbiome, Tight junctions, Transcriptomics, Epithelial cells, Metabolites, Lactobacilli, STIs, HIV

## Abstract

**Background:**

Women with a cervicovaginal microbiota dominated by *Lactobacillus* spp. are at reduced risk of acquiring sexually transmitted infections including HIV, but the biological mechanisms involved remain poorly defined. Here, we performed metaproteomics on vaginal swab samples from young South African women (*n* = 113) and transcriptomics analysis of cervicovaginal epithelial cell cultures to examine the ability of lactic acid, a metabolite produced by cervicovaginal lactobacilli, to modulate genital epithelial barrier function.

**Results:**

Compared to women with *Lactobacillus*-depleted microbiota, women dominated by vaginal lactobacilli exhibit higher abundance of bacterial lactate dehydrogenase, a key enzyme responsible for lactic acid production, which is independently associated with an increased abundance of epithelial barrier proteins. Physiological concentrations of lactic acid enhance epithelial cell culture barrier integrity and increase intercellular junctional molecule expression.

**Conclusions:**

These findings reveal a novel ability of vaginal lactic acid to enhance genital epithelial barrier integrity that may help prevent invasion by sexually transmitted pathogens.

Video abstract

**Supplementary Information:**

The online version contains supplementary material available at 10.1186/s40168-022-01337-5.

## Background

The cervicovaginal microbiota has a substantial influence on sexual and reproductive health in women including their susceptibility to sexually transmitted infections (STIs) [1]. The composition of the cervicovaginal microbiota varies between women of different age, ethnicity and geographical location and is influenced by menstrual cycle phase and behavioural factors [2–4]. A cervicovaginal microbiota dominated by noninflammatory *Lactobacillus* spp. is considered optimal [5] and is associated with favourable health outcomes including lower rates of preterm birth [6–8] and reduced susceptibility to STIs including human immunodeficiency virus (HIV) [1, 9–11]. Women with a cervicovaginal microbiota dominated by *L. crispatus* have a 4.4-fold reduced risk of HIV acquisition compared to women with a nonoptimal microbiota lacking lactobacilli [10]. In contrast, the most common form of a nonoptimal microbiota, bacterial vaginosis (BV), characterised by an increase in anaerobic bacteria and depletion of lactobacilli, is associated with adverse pregnancy outcomes including low birth weight [12], spontaneous preterm birth [7] and increased risk of acquiring and transmitting STIs including HIV [1, 13–16].

The mucosal surface of the female reproductive tract (FRT) is the primary site of entry for many STIs including HIV and, under optimal conditions, possesses substantial physical and immunological defences against pathogen invasion [17]. Interactions between FRT epithelial cells are mediated by intercellular junctional molecules comprising tight junctions, which control paracellular permeability, along with adherens junctions and desmosomal proteins that represent a physical barrier to the passage of microorganisms [18]. This barrier plays an integral role in precluding HIV transmission by preventing virions present in the cervicovaginal lumen from accessing target cells typically located in the subepithelial lamina propria [19, 20].

Invading microorganisms can penetrate the columnar (endocervix and endometrium) and the stratified (ectocervix and vagina) epithelial cell layers through different mechanisms. The FRT epithelial barrier can be compromised by physical damage including microabrasions and/or disruption of the intercellular junctional barrier where the pathogen infiltrates between the intercellular space by paracellular penetration [20, 21]. Alternatively, pathogens can penetrate the epithelium by transcytosis where the apically endocytosed pathogen is transferred via the basal epithelial surface by cell-to-cell spread to susceptible target cells, as reported for HIV in vitro [22]. Paracellular penetration is considered a major mechanism for invasion by pathogens including HIV and has been observed in human cervical explants and following in vivo vaginal exposure of non-human primates [20].

Many microorganisms have evolved strategies to target junctional molecules to aid penetration. Viruses including human papillomavirus [23] and HIV [24, 25] directly modulate the integrity of the FRT epithelial barrier to increase pathogen transmigration and facilitate infection. The increased risk of STIs associated with BV may also be due in part to effects on epithelial barrier integrity, as cervicovaginal fluid from women with BV disrupts the endocervical epithelial barrier and increases transmission of HIV-infected lymphocytes in vitro [26].

Whilst there is an established relationship between an optimal *Lactobacillus*-dominant microbiota and favourable sexual and reproductive health outcomes, the mechanisms through which this occurs are still being uncovered. In addition to precluding replication of STIs [27–29], lactobacilli and their products may act directly on the FRT mucosa to promote beneficial properties including enhanced wound healing [30], epithelial barrier integrity [31] and a noninflammatory state [32, 33]. Whether metabolites produced by vaginal lactobacilli directly enhance the FRT epithelial barrier is unknown.

Lactobacilli produce the metabolite lactic acid (LA) at levels that can vary depending on the species and strain [34, 35]. In women with a microbiota dominated by optimal *Lactobacillus* spp., LA concentrations are approximately 110 mM (1% w/v) which acidifies the vagina to a pH of ~3.5 [4, 36]. LA has direct antiviral and antimicrobial activity against BV-associated bacteria [37], HIV [38, 39] and other STIs including herpes simplex virus 2 [40] and *Neisseria gonorrhoeae* [27]. We have shown that physiologically relevant concentrations of LA have an anti-inflammatory effect on FRT epithelial cells and inhibit pro-inflammatory cytokine and chemokine production elicited by viral and bacterial pathogen-associated molecular patterns [41, 42]. LA can exist in both L- and D-isomers, with different *Lactobacillus* spp. preferentially producing each isoform [34, 43]. The protonated form of LA predominates at a pH below its acid dissociation constant (*pKa* 3.86) and possesses virucidal and anti-inflammatory activity, in contrast to the lactate anion that predominates at pH > 3.86 [38, 41, 42]. These data, and others [29, 44, 45], indicate that metabolites produced by optimal FRT microbiota species are likely key mediators of the health benefits associated with *Lactobacillus*-dominant microbiota.

There is limited evidence that cervicovaginal bacteria and their metabolites can enhance FRT epithelial integrity. Zevin et al. identified an association between *Lactobacillus*-dominant FRT microbiota and expression of host proteins relevant for maintaining epithelial barrier function, observing a positive association observed between levels of bacterial L-lactate dehydrogenase (L-LDH, a key enzyme involved in LA production by lactobacilli) and epithelial barrier proteins [46]. We considered whether these observations indicated a role for vaginal LA in directly enhancing the FRT epithelial barrier and whether this may be mediated by modulation of intercellular junctional molecules. In this study, we explored the in vivo relationship between LA and epithelial barrier integrity in a large cohort of young women in South Africa with diverse FRT microbiota and used functional and transcriptomic in vitro analyses to demonstrate the ability of LA to directly enhance FRT epithelial barrier integrity.

## Methods

### Metaproteomics analysis of cervicovaginal swab samples

Analysis of the vaginal microbiome and secretome was performed on cervicovaginal fluid samples collected from young women in the Women’s Initiative in Sexual Health (WISH) study conducted in South Africa. A full description of the study design and cohort have been detailed previously [47] but included sexually active, HIV-negative, nonpregnant women aged 16–22 years. Lateral vaginal wall swabs were collected by the study nurse by rotating Dacron swabs 360° on the lateral vaginal wall before being placed in 1 ml phosphate-buffered saline (PBS), transported to the laboratory at 4° C and stored at −80 °C. Samples were computational randomised and the eluted material subjected to metaproteomic analysis using liquid chromatography-tandem mass spectrometry (LC-MS/MS) on a Q-Exactive quadrupole-Orbitrap MS (Thermo Fisher Scientific, MA, USA) coupled with a Dionex UltiMate 3000 nano-UPLC system (120 min per sample) [45]. Proteomic data were used to infer vaginal microbiome composition and host secretome as described previously [45]. Briefly, proteins were identified using two different databases: (i) UniProt database restricted to human and microbial entries (73,910,451, release August 2017) and filtered using the MetaNovo pipeline [48] and (ii) human proteins combined with a vaginal metagenome-based database obtained from the study of Afiuni-Zadeh et al. [49]. Taxonomy was assigned using UniProt, and relative abundance of each taxon was determined by aggregating the intensity-based absolute quantification (iBAQ) values of all proteins identified for each taxon. The taxonomic analysis of the proteins identified using both of the databases was similar; however, the first database resulted in the identification of a greater number of proteins compared to the vaginal metagenome database. The first database also showed a high degree of similarity compared to 16S rRNA gene sequence data from a subset of the same women and was thus used for downstream analysis [45]. As the majority of taxa identified had ≤ 2 proteins detected, a more stringent cut-off was applied to include only taxa with ≥ 3 detected proteins or 2 proteins detected in multiple samples. For LDH analysis, log_2_-transformed iBAQ values for bacterial L-LDH and D-LDH were aggregated and the participants categorized into low (< median) or high (≥ median) groups. The presence of BV was assessed by Nugent score (Nugent BV) [5], with a score ≥ 7 considered as BV, 4–6 as an intermediate and ≤ 3 as non-BV [50]. This study evaluated samples from 113 women recruited from the Cape Town study site who had sufficient sample and metadata available for analysis.

The limma R package [51] was then used to identify differentially abundant host proteins between these categories. Logistic regression was used to adjust for potential confounders including *Lactobacillus* spp. relative abundance, STIs, prostate-specific antigen and contraceptives.

### Culture and LA treatment of cervicovaginal epithelial cells

The human ectocervical (Ect1/E6E7) and vaginal (VK2/E6E7) cell lines (purchased from the ATCC) were cultured and seeded for treatment on transwell supports as previously described [41]. Average LA concentration in cervicovaginal fluid is 1% (+/−0.2%) [36]; however, the multilayered structure of the epithelium and the presence of mucus means viable epithelial cells in basal layers are likely to be exposed to concentrations < 1%. Indeed, previous optimisations indicated 0.3% LA is the highest concentration tolerated by monolayers of cervicovaginal epithelial cells without eliciting cytotoxicity [41] and was thus utilised for these analyses. Cells were treated for 1 h with media containing 0.3% L-LA or D-LA (Sigma-Aldrich) at either pH 3.9 or 7 or low pH media control (pH 3.9, media acidified by hydrochloric acid, HCl) added to the apical compartment. HCl-containing media was replenished during the 1-h treatment to maintain a pH < 4.5 [41]. Following treatment, media was removed and cells washed with calcium and magnesium-free PBS (PBS−, Life Technologies, Carlsbad, CA). Fresh media was added before returning to culture for either a further 4 h (for RNA analysis) or 24 h (for all other experiments). Epithelial barrier integrity was assessed by transepithelial electrical resistance (TEER) using a Millicell-ERS Voltohmmeter and probe (Millipore, MA). The unit area resistance (*Ω* × cm^2^) was calculated by multiplying the sample resistance by the membrane area.

### Impact of LA treatment on epithelial barrier gene expression in FRT epithelial cells

RNA was extracted using the RNeasy mini kit (Qiagen, Hilden, Germany) as previously described [41]. To determine gene expression in LA-treated cervicovaginal epithelial cells, next-generation RNA-Seq analysis was performed at the Australian Genome Research Facility (AGRF, Melbourne, Australia) using the HiSeq 2500 NGS platform (Illumina, San Diego, CA) and an Illumina bcl2fastq 2.20.0.422 pipeline with an output of 100 bp paired-end reads. Sequence read datasets were checked for quality using FastQC [52]. Reads were trimmed based on quality scores using the Trim Reads tool with default parameters within CLC Genomics Workbench 11.0 (CLC; Qiagen). Overlapping paired reads were merged, and then, all reads were mapped against the *Homo sapiens* reference genome (build version hg38) using the RNA-Seq tool in CLC. Differentially expressed gene (DEGs) analysis was performed in Degust (http://degust.erc.monash.edu/), using the voom/limma method [53, 54]. A false discovery rate (FDR) cut-off of < 0.05 and a Log_2_ fold change (FC) ≥ 0.5 were used to identify genes with significantly altered expression. Targeted interrogation of the expression of genes related to the tight junction barrier was performed using a gene list derived from the RT^2^ Profiler™ Human Tight Junctions PCR Array (Qiagen). To identify and visualise significantly overrepresented gene ontologies among the DEGs, we utilised the BiNGO application [55] within Cytoscape v.3.8.2 [56] to perform a hypergeometric test using the Benjamini and Hochberg FDR correction. Unranked lists of DEGs were tested against the July 02, 2021 Gene Ontology release (http://geneontology.org) [57].

Altered expression of junctional molecules was confirmed by qRT-PCR using RT^2^ SYBR Green Mastermix (Qiagen) and primers specific for the following tight junction factors and housekeeping genes; claudin-1 (*CLDN1*, Cat no. PPH02779A), claudin-4 (*CLDN4*, Cat no. PPH07330D), occludin (*OCLN*, Cat no. PPH02571B), junctional adhesion molecule A (*F11R*, Cat no. PPH02605A), zona occludens 2 (*TJP2*, Cat no. PPH09978B) and the housekeeping genes glyceraldehyde 3-phosphate dehydrogenase (*GAPDH*, Cat no. PPH00150F), hypoxanthine phosphoribosyltransferase 1 (*HPRT1*, Cat no. PPH01018C) and ribosomal protein lateral stalk subunit P0 (*RPLP0*, Cat no. PPH21138F; all from Qiagen). Amplification was performed with a thermocycle of 95 °C for 10 min, 40 cycles of 95 °C for 15 s and 40 °C for 60 s, followed by melt curve analysis. Primer specificity was verified by melt curve analysis, which indicated a single amplification product with a unique melting temperature for each gene target. Ct values were analysed to determine relative gene expression, which was standardised to the average expression of the housekeeping genes and calculated using the 2^−ΔΔCT^ method [58].

### Analysis of tight junction protein expression and localisation

Levels of tight junction proteins in ectocervical epithelial cells were determined by Western blot using primary antibodies against claudin-1, claudin-4, tight junction protein (TJP) 1 and TJP2 (Cat no. 51-9000, 36-4800, 33-9100 and 71-1400, respectively, all from Thermo Fisher Scientific), β-actin (Cat no. ab8224, Abcam) and secondary antibodies Alexa Fluor 680-goat anti-mouse IgG (Thermo Fisher Scientific) and Alexa Fluor 800-donkey anti-rabbit IgG (Li-COR, Lincoln, NE). Proteins were visualised with an Odyssey Infrared Imaging system and analysed with Image Studio Lite software (both from Li-COR). Protein levels were normalised to β-actin protein intensity.

### Immunofluorescence and confocal microscopy

Claudin-4 protein location in Ect cells was visualised by immunofluorescence and confocal microscopy. Epithelial cells were fixed with ice-cold methanol, rehydrated with 1% foetal bovine serum (FBS) in PBS− and permeabilised with 0.2% Triton X-100 in PBS− for 10 min. After blocking with 3% BSA in PBS−/0.2% Triton X-100, cells were incubated with rabbit polyclonal anti-claudin-4 antibody (Invitrogen, Cat no. 36-4800, 1:25 dilution) and then Alexa Fluor 488-goat anti-rabbit IgG secondary antibody (Invitrogen, Cat no. A-11008, 1:200). Nuclei were visualised using Hoechst 33342 stain (Thermo Scientific). Transwell membranes and attached cells were excised and mounted on slides with ProLong Gold mounting medium (Invitrogen). Confocal Z-stacks were captured at 0.2 μm per section, 25 sections in total using a Nikon A1R confocal microscope (Nikon Corporation, Japan) at 60× magnification with an oil-immersion lens. Images were selected at random (at least 3 stacks per slide) and captured for quantitative analysis with ImageJ software (National Institute of Mental Health). Images were thresholded to eliminate background fluorescence. The sum of fluorescence intensity was calculated for the stack, and mean fluorescence intensity (MFI) was determined.

### Transcriptomic analysis of vaginal epithelial cells exposed to bacterial culture supernatants

Bacterial culture supernatants were generated by inoculating 10 ml of culture media with 1 ml of 1 × 10^7^ colony-forming units (CFU) of *L. crispatus* (ATCC 33197), *L. jensenii* (ATTC 25258), *L. iners* (ATTC 55195) or *G. vaginalis* (ATCC 14018). NYC-III media was used for propagation of *L. crispatus*, *L. jensenii* and *L. iners*, whilst Tryptic Soy Broth (TSB) was used for *G. vaginalis*. CFU to OD_600_ measures were pre-calculated for each strain. Cultures were grown anaerobically for 48 h to late stationary phase. *L. crispatus*, *L. jensenii*, *L. iners* and *G. vaginalis* reached 5.2 × 10^8^ CFU/ml, 5.8 × 10^8^ CFU/ml, 4.1 × 10^8^ CFU/ml and 1.2 × 10^9^ CFU/ml, respectively. The 10 ml of culture supernatant was clarified by centrifugation at 3000 × *g* for 10 min, sterile filtered (0.2 μm filter) and stored at −20 °C until use. Bacterial supernatants were diluted to 20% (v/v) in complete VK2 cell culture medium and added to VK2 cells for 13 h. VK2 cells were serum starved for 24 h in keratinocyte serum-free base media only, prior to addition of 20% bacterial culture supernatants. After the exposure, media was removed, cells were washed once with 1× PBS and 300 μl RNAlater (QIAGEN) was added to wells. Cells were mechanically detached and stored at −80 °C before total RNA extraction. Total RNA was extracted using the MasterPure™ Complete DNA and RNA purification kit (Lucigen, WI, USA) according to manufacturer’s instructions. Methodology for transcriptomic analyses by RNASeq including library preparation, RNA sequencing and read mapping has been detailed previously [29]. LA concentration within bacterial supernatant-containing media was quantified by using the D/L lactic acid assay kit (R-Biopharm AG, Darmstadt, Germany, Cat no. 11112821035). Concentration of the protonated form of LA within treatment media was calculated from the sample pH and LA concentration using the Henderson-Hasselbalch equation as previously described [36]. Total lactate and protonated LA were normalised to the final CFU/ml for *L. crispatus*, *L. jensenii*, *L. iners* and *G. vaginalis* achieved at the stationary phase.

## Results

### Bacterial lactate-dehydrogenase relative abundance is increased in cervicovaginal secretions from women with *Lactobacillus*-dominated microbiota

**Fig. 1:**
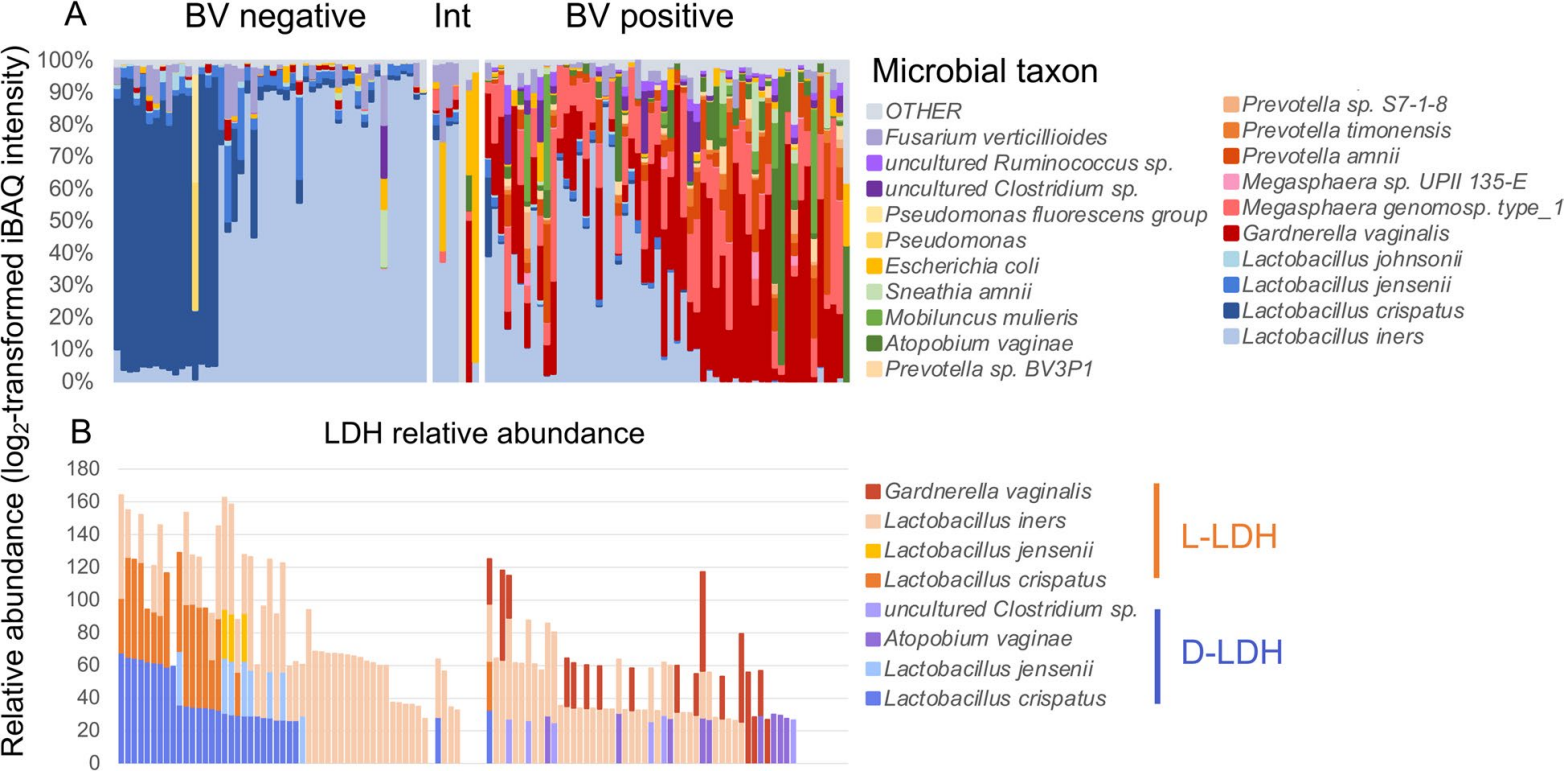
Higher relative abundance of bacterial LDH in women with a *Lactobacillus*-dominated microbiome. The vaginal microbiome composition of 113 young women from Cape Town, South Africa, was determined using liquid chromatography-tandem mass spectrometry, and participants were classified as being bacterial vaginosis (BV) negative, positive, or intermediate (Int) using Nugent-BV criteria. Proteins were identified using MaxQuant, and a custom database was generated using de novo sequencing to filter the UniProt database. Taxonomy was assigned using UniProt, and relative abundance of each taxon was determined by aggregating the intensity-based absolute quantification (iBAQ) values of all proteins identified for each taxon. The relative abundance of the 20 most abundant bacterial species is indicated for each participant in (**A**). The relative abundance of L- and D-lactate dehydrogenase (LDH) protein derived from various bacterial sources was measured in vaginal secretions using metaproteomic analyses for each participant (**B**)

A previous proteomic analysis of cervicovaginal secretions from 41 women identified an increased relative abundance of cornified envelope factors (INVO and SPR1A), which are involved in maintaining epithelial barrier integrity, in women with a *Lactobacillus-*dominated as compared to a *G. vaginalis*-dominated microbiota [46]. Furthermore, levels of lactate dehydrogenase (LDH, a key enzyme in LA synthesis responsible for catalysing the conversion of pyruvate to lactate) from *L. iners* were positively associated with these factors. We therefore explored the relationship between LA isomers produced from different *Lactobacillus* spp. and proteins specifically involved in epithelial barrier integrity in vivo. We conducted a proteomic analysis utilising cervicovaginal fluid samples collected from 113 HIV-uninfected, nonpregnant women enrolled in the WISH study in Cape Town, South Africa (Table 1). Half of these women were Nugent-BV positive (50%, Table 1), whilst the remainder typically exhibited a microbiota dominated by *L. iners* or *L. crispatus* (Fig. 1A). LC-MS/MS analysis detected 3186 different peptides, 55% of which were of bacterial origin, and 8 distinct LDH proteins produced from bacterial species were identified in these samples (Fig. 1B). Bacterial LDH abundance was analysed as an indicator of LA levels given our previously findings that LDH relative abundance produced by clinical *Lactobacillus* isolates correlates with lactate concentrations in vitro [59]. High levels of host-derived LDH, which has only 39% sequence homology with LDH derived from gram-positive bacteria [60], were also detected but were excluded from the analysis. As expected, bacterial LDH protein was substantially less abundant in cervicovaginal samples from women with BV as compared to those with a microbiota dominated by *Lactobacillus* spp. (Fig. 1). Cervicovaginal secretions from women with a microbiota dominated by *L. iners* contained primarily L-LDH, whilst both L- and D-LDH were detected in women with *L. crispatus*-dominated microbiome (Fig. 1B), consistent with the known differential production of LA isomers by these species [34, 43]. *L. iners* was detected in a portion of women with Nugent BV at a relative abundance of up to 86% (Fig. 1A), and in these women, *L. iners*-derived L-LDH was also detected. Consistent with previous findings [2, 61], *L. crispatus* was rare in women with BV, and consequently, minimal D-LDH was detected.

**Table 1:** Demographic and clinical characteristics of cohort

	***n*** (%)
Black race	113 (100)
Median age in years (range)	18 (16–22)
*Sexually transmitted infections/sexual activity*	
*Chlamydia trachomatis*	37 (33)
*Neisseria gonorrhoeae*	6 (5)
*Trichomonas vaginalis*	6 (5)
*Mycoplasma genitalium*	7 (6)
Herpes simplex virus-2 (PCR positive)	4 (4)
PSA positive	21 (19)
*Microbiome features*	
Yeast cells detected	11 (10)
Nugent-BV positive	56 (50)
Nugent-BV intermediate	7 (6)
*Contraceptive use*	
Using DMPA	20 (18)
Using Nur-Isterate	78 (70)
Using OCP	6 (5)
Using Implanon	8 (7)
Using NuvaRing	1 (1)

We found that total bacterial LDH relative abundance correlated inversely with cervicovaginal pH (Spearman’s Rho = −0.44; *p* < 0.001; Supplementary Fig. 1), which agrees with previous observations [46] and is consistent with the known effect of LA in maintaining low vaginal pH [36]. In contrast, host L-LDH showed a weak positive association with vaginal pH (Spearman’s Rho = 0.265, *p* = 0.007; data not shown). Taken together, these data indicate a higher abundance of bacterial-derived LDH in cervicovaginal samples from women with *Lactobacillus*-dominated microbiota where vaginal LA levels are known to be high and pH levels low.

### High LDH relative abundance is associated with increased expression of epithelial barrier proteins in vivo

**Fig. 2:**
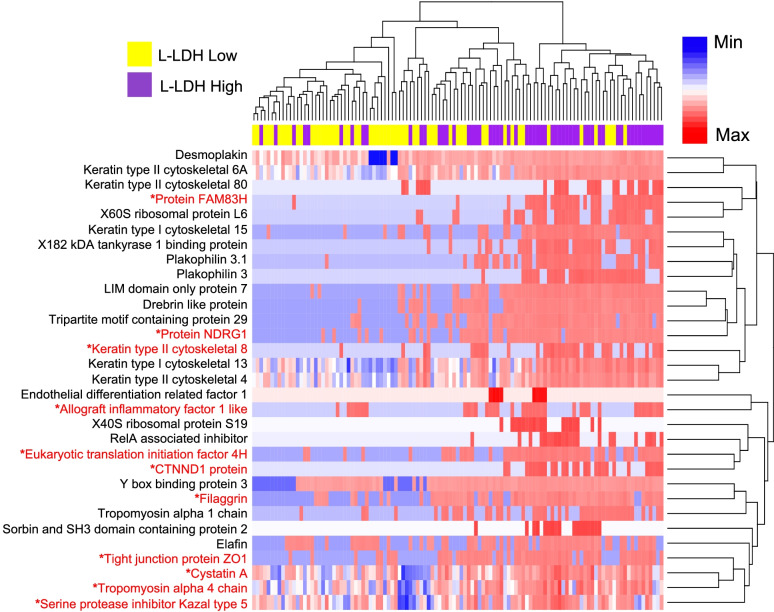
L-LDH relative abundance in the FRT is associated with expression of epithelial junction proteins. Unsupervised hierarchical clustering of intensity-based absolute quantification (iBAQ) values for epithelial barrier-related proteins that were found to be differentially abundant between women (*n* = 113) with levels of L-LDH protein above (high, purple) or below (low, yellow) the median value for the cohort. The moderated *t*-test (limma package, R) was used to identify proteins significantly associated with L-LDH relative abundance following false discovery rate adjustment for multiple comparisons. *Proteins that remained significantly associated with L-LDH after adjusting for *Lactobacillus* relative abundance, as well as the presence of sexually transmitted infections, contraceptive use, and detection of prostate-specific antigen

To examine the relationship between bacterial LDH abundance and host protein expression, we stratified women into high and low bacterial LDH categories based on the median relative LDH abundance of the cohort respectively. An analysis of host protein relative abundance identified 96 proteins which showed a significant differential abundance between high and low L-LDH groups (FDR adjusted *p* < 0.05; Supplementary Table 1), with 31 of these proteins (32%) having epithelial barrier function-related gene ontologies (Fig. 2). Although detection of *L. crispatus*, and consequently D-LDH protein, was lower in this cohort, 4 proteins were differentially abundant between women with high and low D-LDH (FDR adjusted *p* < 0.05; Supplementary Table 1). These included two proteins with gene ontology terms related to epithelial barrier function: keratin 8 and dermokine. Logistic regression analysis was performed to assess the relationship between L-LDH groups and expression of these epithelial barrier-associated proteins after adjusting for multiple potential confounders including *Lactobacillus* relative abundance, the presence of STIs, contraceptive use, and detection of prostate-specific antigen (PSA, an indicator of recent vaginal intercourse). We found that 11 of the 31 proteins remained significantly associated with L-LDH abundance, including tight junction protein 1 (TJP1/ZO-1; Fig. 2 and Supplementary Table 2). Similarly, 10 of 31 proteins remained significantly associated with L-LDH abundance following adjustment for BV status, STIs, contraceptive use and PSA. To account for protease production by nonoptimal bacteria that may contribute to epithelial barrier degradation and dysfunction, we aggregated the relative abundance of the 23 proteins with protease/peptidase activity produced by *G. vaginalis*, *Megasphaera* spp., *Mobiluncus mulieris*, *Prevotella* spp. and *Sneathia amnii* that were detected in our samples. Whilst total protease/peptidase relative abundance was inversely associated with epithelial barrier proteins, when we evaluated the association between L-LDH and epithelial barrier proteins, adjusting for protease/peptidase relative abundance, we found that 30/31 remained significantly associated with L-LDH. These analyses provide in vivo evidence of an association between cervicovaginal relative abundance of the key enzyme responsible for LA production and levels of epithelial junctional proteins that govern barrier integrity.

### Lactic acid treatment of ectocervical epithelial cells enhances epithelial barrier integrity

**Fig. 3:**
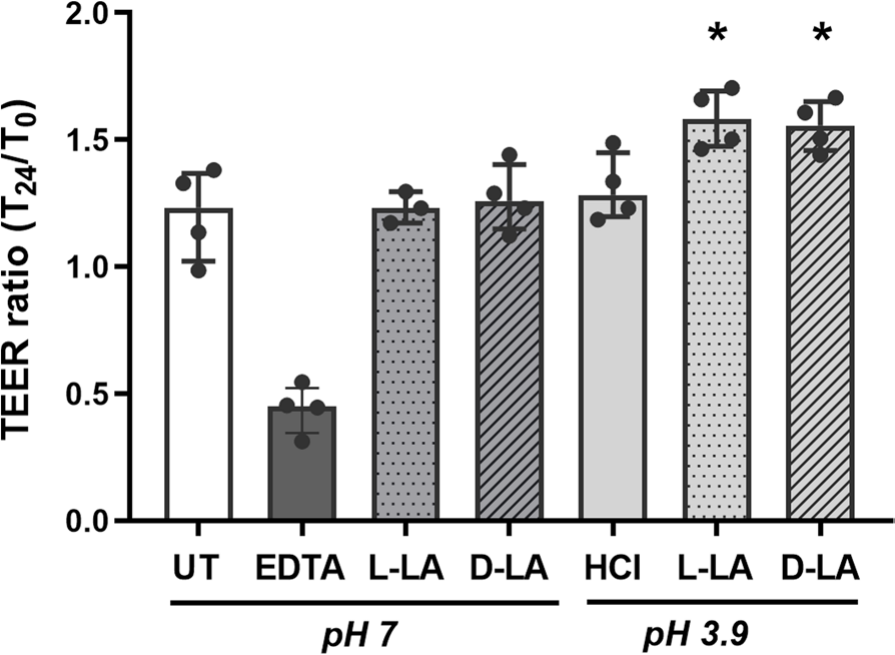
Protonated LA increases the barrier integrity of ectocervical epithelial cells. Ectocervical cells (Ect1) seeded into transwells were apically treated for 1 h with media alone (untreated, UT) or media containing 0.3% L- or D-lactic acid (LA) at either low (pH 3.9) or neutral (pH 7) pH, low pH media alone (HCl, pH 3.9), or 2 mM EDTA. Epithelial barrier integrity was assessed by transepithelial electrical resistance (TEER) measured at baseline (T0) and 24 h after treatment (T24) expressed as the TEER ratio (T24/T0). Graph shows median and interquartile range for each condition from 4 independent experiments. *p < 0.05 compared to untreated control, as determined by Mann-Whitney *U*-test

Given the in vivo observations supporting an association between LA and cervicovaginal epithelial integrity, we assessed whether LA produced by vaginal *Lactobacilli* spp. was able to directly enhance epithelial barrier integrity in vitro. As discussed above, lactobacilli can produce both L and D isomers of LA dependent on species, with the biologically active protonated form of LA predominating at pH below 3.9 [38, 41, 42]. Ectocervical epithelial cells were therefore apically treated with a physiologically relevant concentration of 0.3% L- or D-LA at pH 3.9 and the change in epithelial barrier integrity assessed by TEER. Treatment of cells with either isoform of LA at pH 3.9 elicited a significant enhancement of barrier integrity as compared to untreated cells (Fig. 3), which was not observed when cells were treated with media acidified to the same pH with HCl. Ethylenediamine tetraacetic acid (EDTA) served as a positive control for barrier disruption in these experiments and elicited an expected decrease in TEER. Similar to our previous findings regarding the pH dependency of LA’s bioactivity [38, 41], no effect on TEER was observed when L- and D-LA containing media were neutralised to pH 7.0, indicating the epithelial barrier-enhancing effect of LA is mediated by the protonated form and not the lactate anion.

### LA elicits altered expression of genes related to epithelial barrier integrity

**Fig. 4:**
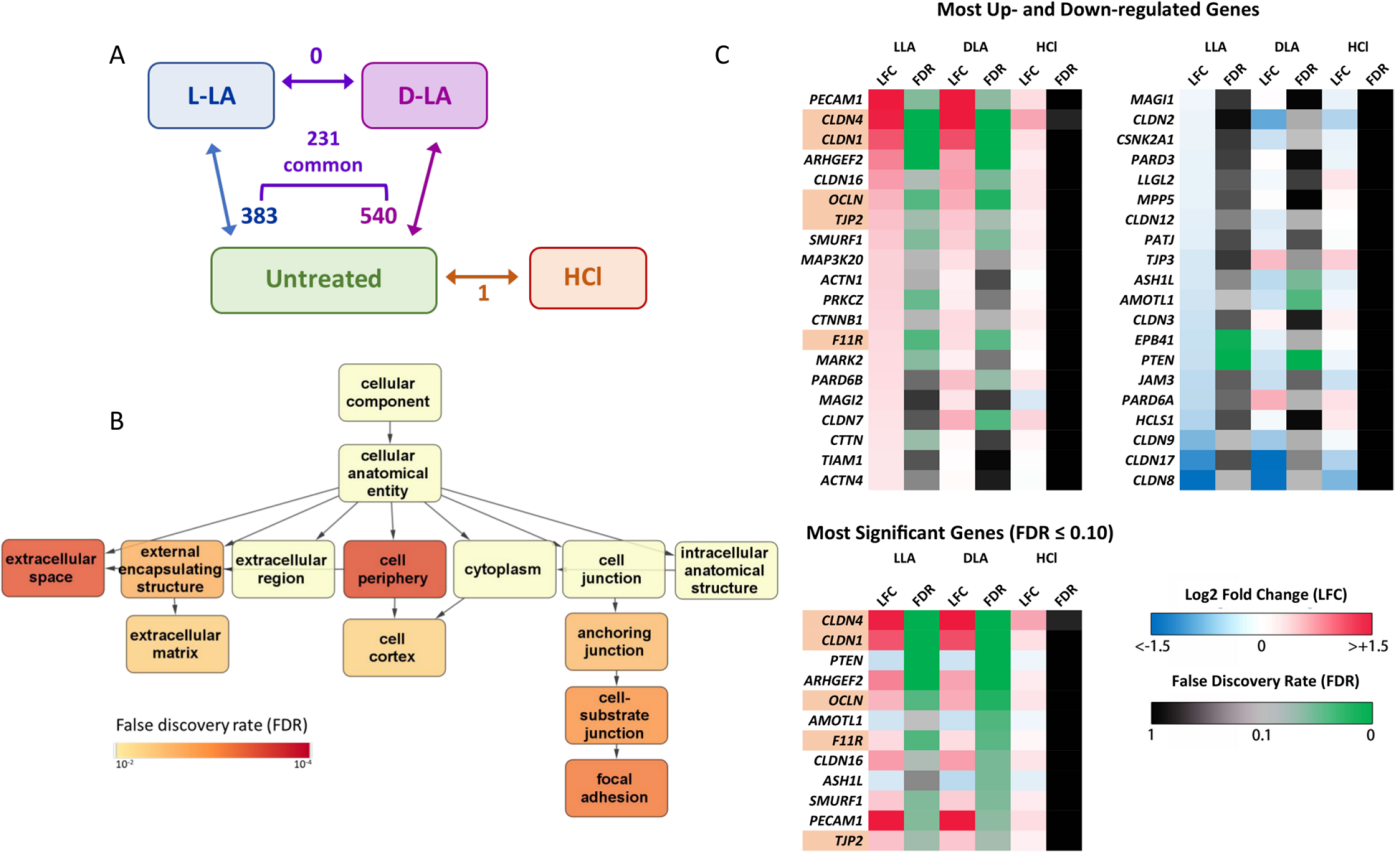
LA treatment of ectocervical epithelial cells increases the expression of genes relevant to barrier integrity. Ectocervical epithelial cells were apically treated with 0.3% L- or D-lactic acid (LA) or low pH media alone (HCl) for 1 h and transcriptomic changes assessed at 4-h post treatment by RNA-Seq. **A** Number of genes differentially expressed between the treatments is shown next to arrows indicating comparison groups as assessed by voom-limma analysis (Log2 fold change > 0.5, false discovery rate [FDR] < 0.05). There were 231 genes which were differentially expressed by both L-LA and D-LA as compared to untreated cells. **B** Gene ontologies enriched by LA treatment. For visual simplicity, ontologies enriched with a *FDR* < 0.01 are shown; all ontologies enriched with a *FDR* < 0.05 are shown in Supplementary Table 2. **C** Expression of genes related to epithelial tight junctions relative to untreated cells ranked by magnitude of up- or downregulation (left panel) or adjusted *p*-value (FDR). Genes highlighted in orange were those selected for further characterisation

To identify the mechanisms of LA-mediated barrier enhancement, we performed transcriptomic analysis of ectocervical epithelial cells treated with L- and D-LA (pH 3.9) using RNA-Seq. This analysis revealed 383 and 540 DEGs between untreated cells and those treated with either L-LA or D-LA, respectively (Fig. 4A and Supplementary Table 3). In contrast, treatment of ectocervical cells with media acidified to pH 3.9 with HCl had negligible effect on gene expression, with only one gene (*HSPA6*) found to be significantly differentially expressed compared to untreated cells (orange box in Fig. 4A). These data suggest that changes to gene transcription elicited by LA are unlikely to be due to low pH alone. No significant differences were observed in DEGs detected between L- and D-LA-treated cells using our stringent analysis criteria (upper boxes, Fig. 4A), suggesting that both isomers of LA mediate an analogous effect on the transcriptome of ectocervical epithelial cells, consistent with their similar effects on increasing epithelial barrier integrity (Fig. 3). Indeed, of the 383 DEGs elicited by L-LA treatment, 231 (60.3%) of the same genes were also significantly altered by D-LA treatment (Fig. 4A). This was further supported by the clustering of L- and D-LA-treated samples on a multidimensional scaling (MDS) plot (Supplementary Fig. 2). These data indicate treatment with L- and D-LA, but not low pH alone, elicits substantial gene changes to immortalized ectocervical cells, and that overlapping transcriptomic signatures are observed with both isomers of LA.

Gene ontology analysis was performed to investigate pathways which were targeted by LA treatment. This revealed that LA treatment altered a broad range of cellular components, functions and processes (Fig. 4B and Supplementary Table 4) with 32/334 (9.6%) of pathways that were significantly enriched by L-LA treatment being associated with barrier function (Supplementary Table 4). A similar proportion of pathways enriched by D-LA treatment were also barrier function related (15/191; 7.9%). To assess the impact of LA treatment on epithelial tight junctions specifically, we separately evaluated expression of key genes known to encode critical tight junction proteins and found a substantial portion of these factors were significantly altered by LA treatment (Fig. 4C and Supplementary Table 5). The most significantly DEGs included those with established roles in facilitating tight junctions including *CLDN1*, *CLDN4*, *OCLN*, *TJP2* and *F11R* (Fig. 4C).

**Fig. 5:**
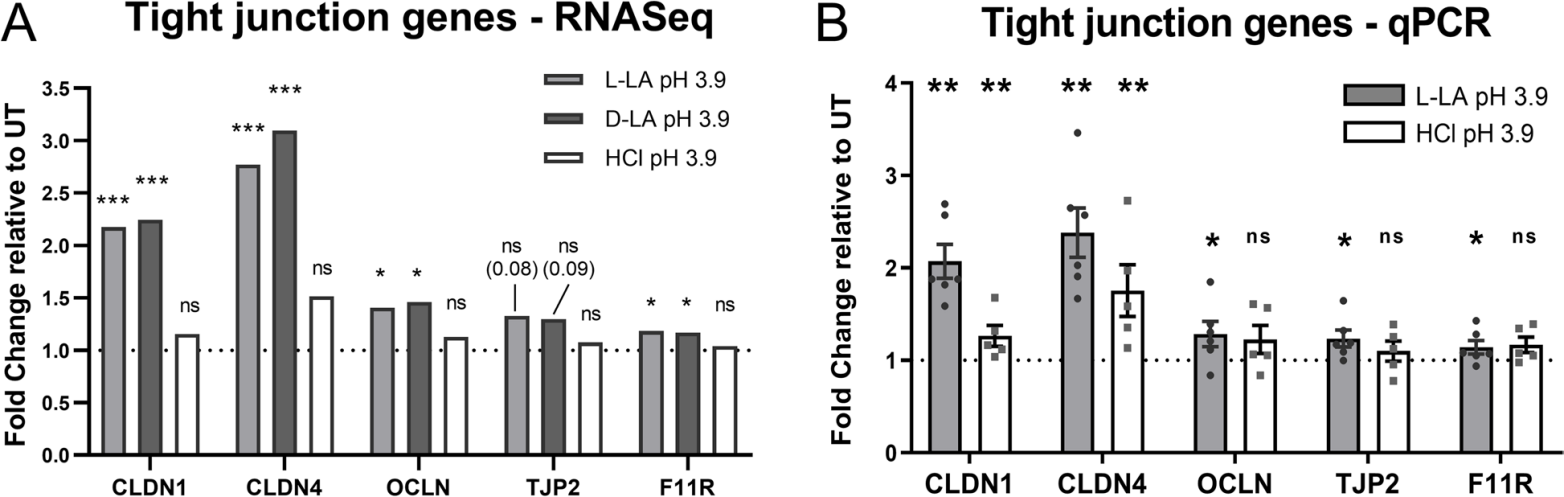
qRT-PCR analysis of expression of tight junction genes in ectocervical cells following L-LA treatment. Ectocervical epithelial cells were apically treated with 0.3% L-lactic acid (L-LA) or low pH media alone (HCl) for 1 h, and gene expression of tight junction factors claudin-1 (CLDN1), claudin-4 (CLDN4), occludin (OCLN), zona occludens-2/tight junction protein-2 (TJP2) and F11R was assessed at 4 h post treatment by RNA-Seq (**A**) or qRT-PCR (**B**). **A** Fold change in expression of tight junction genes in ectocervical epithelial cells treated with L-LA, D-LA or HCl-treated cells relative to untreated, as determined by RNA-Seq analysis. * and *** represent false discovery rate < 0.05 and < 0.001, respectively. **B** Relative expression (fold change) of tight junction genes compared to untreated cells (indicated by dotted line) as determined by qRT-PCR. Graph shows mean +/− SEM from *n* = 5–6 independent experiments. * and ** represent *p* < 0.05 and < 0.01, respectively, compared to untreated cells as determined by the Mann-Whitney *U*-test. NS, not significant

To validate the differential expression of tight junction genes by LA treatment observed in the RNA-Seq analysis, we performed qRT-PCR analysis of a subset of genes in ectocervical cells treated with LA (pH 3.9) under the same conditions used to generate the RNA-Seq data. Given the similar impact of both L- and D- LA on transcriptional changes observed above, we limited the analysis to L-LA for these validation experiments. Consistent with the RNA-Seq data, qRT-PCR analysis demonstrated significantly increased expression of *CLDN1*, *CLDN4*, *OCLN* and *F11R* following LA treatment (Fig. 5, *p* < 0.05 for all), whilst expression of *TJP2* was also significantly increased by L-LA in the qRT-PCR analysis. Interestingly, qRT-PCR analysis also showed increased expression of *CLDN1* and *CLDN4* due to low pH alone, but the magnitude of this effect was lower than that observed for L-LA (Fig. 5). We also analysed expression of the two most significant DEGs, *CLDN1* and *CLDN4*, in primary cervicovaginal cells and observed a similar effect of L-LA on expression as in ectocervical cells (Supplementary Fig. 3). Taken together, these molecular analyses indicate that physiological concentrations of both L- and D-LA at pH 3.9 upregulate the expression of genes involved in cell barrier and tight junction formation which likely contributes to enhanced barrier function of ectocervical epithelial cells.

### LA upregulates the expression of tight junction proteins claudin-1 and claudin-4

**Fig. 6:**
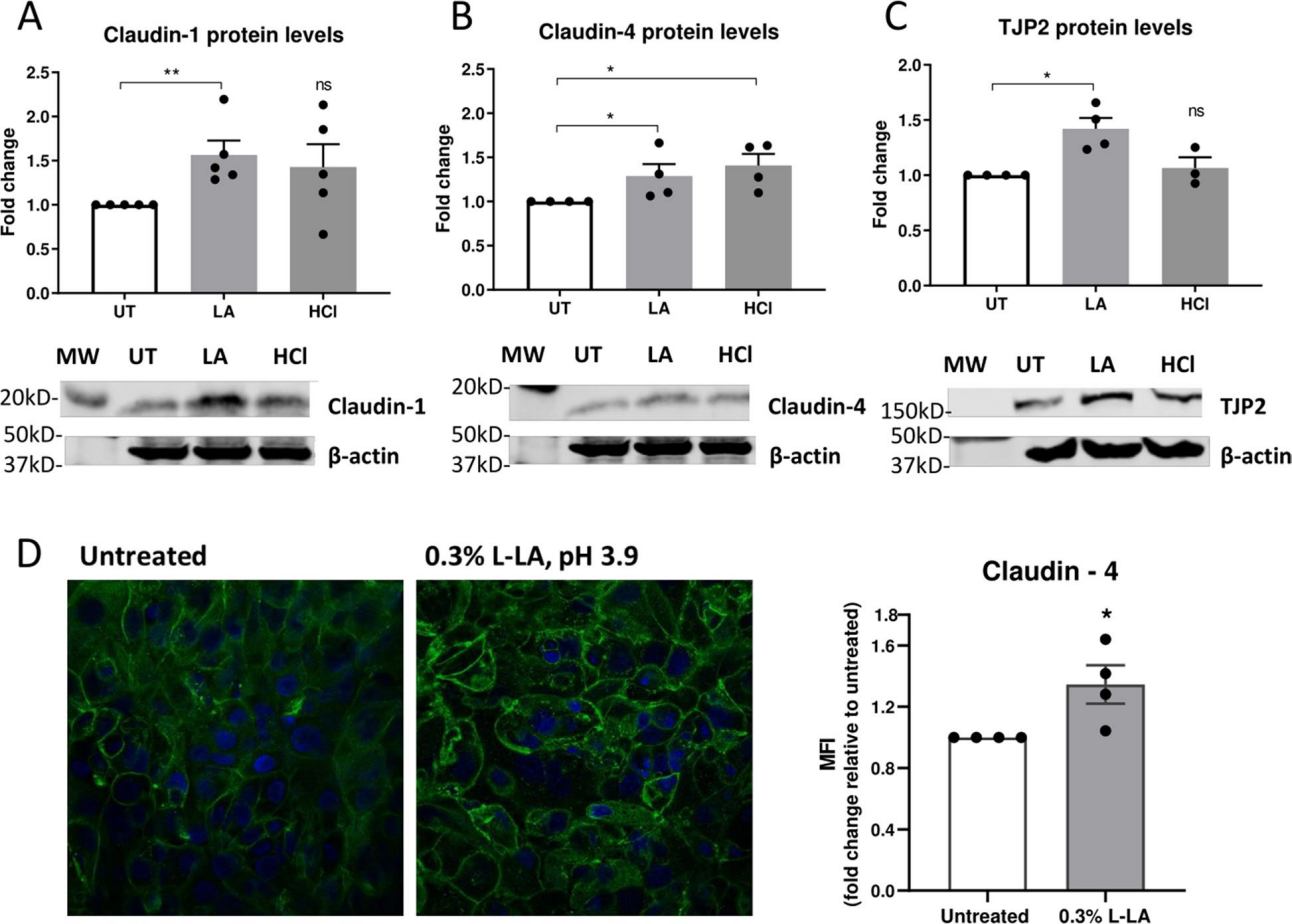
Increased expression of tight junction proteins in ectocervical cells by L-LA. Protein levels of barrier proteins claudin-1 (**A**), claudin-4 **(B**) and tight junction protein-2 (TJP2) (**C**) in ectocervical epithelial cells stimulated apically with 0.3% L-LA pH 3.9 (LA) or low pH media (HCl, pH 3.9) for 1 h followed by lysis of cells at 24 h and assessed by Western blot analysis. Target protein levels were standardised to β-actin in the same sample. **D** Expression of claudin-4 was visualised in ectocervical epithelial cells treated as above by immunofluorescence and confocal microscopy. Images from a single experiment representative of *n* = 4 replicates are shown (left), and mean fluorescence intensity (MFI) of claudin-4 signal intensity was assessed using ImageJ software and expressed relative to untreated samples. All graphs show mean +/− SEM (expressed as fold change compared to untreated cells) from 3 to 5 independent experiments. Significance was assessed by the Mann-Whitney *U*-test; *, **p < 0.05 and 0.01, respectively

To confirm the upregulation of tight junction gene expression was associated with increased levels of tight junction proteins, ectocervical cells were treated with 0.3% L-LA pH 3.9 and levels of the most highly expressed factors claudin-1 and -4 assessed by Western blot. Consistent with the gene expression data, protein levels of both claudin-1 and -4 were significantly higher in L-LA-treated cells as compared to untreated cells (Fig. 6A and B, *p* = 0.01 and 0.03, respectively), and higher levels of claudin-4 were also observed in cells treated with low pH media alone (Fig. 6B). Protein levels of TJP2/ZO-2 were also significantly increased in L-LA-treated cells (Fig. 6C), which was consistent with the significantly increased expression of *TJP2* found in the qRT-PCR analysis. Finally, to confirm that the protein levels observed in Western blot reflected relevant proteins localised at regions of cell-cell contact, we analysed ectocervical cells by fluorescence microscopy and observed focussed localisation of claudin-4 at intercellular regions in ectocervical cells treated with L-LA and a significantly increased intensity of staining for claudin-4 in L-LA-treated cells as compared to untreated samples (Fig. 6D). Thus, LA acts directly on FRT epithelial cells to increase the expression of junctional proteins at sites of cell-cell attachment, which likely contributes to strengthening of the epithelial barrier.

### Bacterial supernatants containing LA increase expression of tight junction genes

**Fig. 7:**
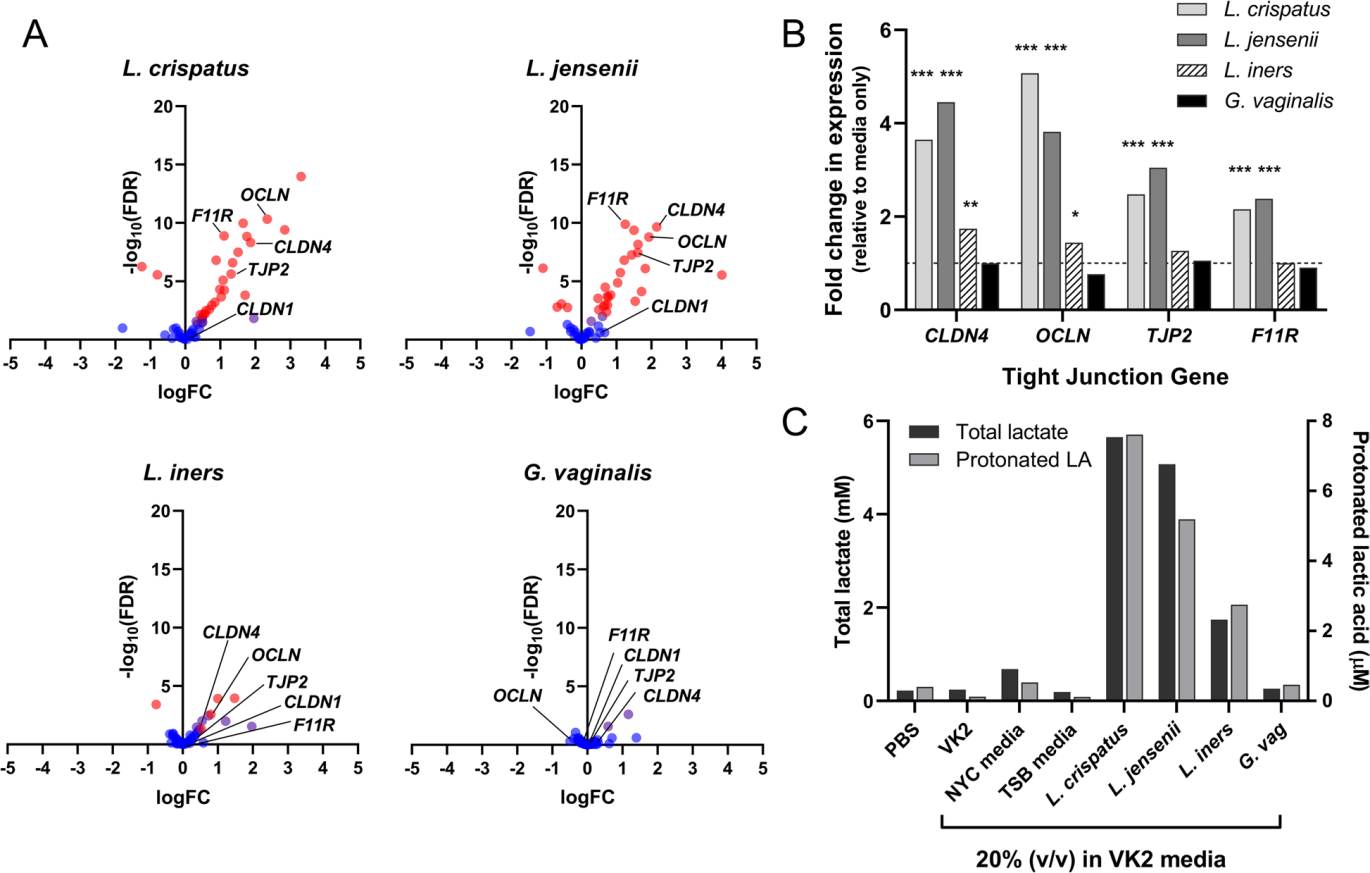
*Lactobacillus* cultures producing high levels of LA upregulate tight junction gene expression. VK2 epithelial cells were treated with media containing 20% (v/v) filtered supernatant from cultures of *L. crispatus*, *L. jensenii*, *L. iners* or *G. vaginalis* bacteria. The impact of treatment on expression of key tight junction genes was analysed by RNA-Seq, and volcano plots of changes in expression following treatment are shown in (**A**), with red dots indicating significantly DEGs (*FDR* < 0.05). **B** Fold change in expression of claudin-4 (*CLDN4*), occludin (*OCLN*), zona occludens-2/tight junction protein-2 (*TJP2*) and *F11R* genes relative to cells treated with media alone (mean, from *n* = 3 replicates; *, ** and ***false discovery rate < 0.5, 0.01 and 0.001, respectively). **C** Concentration of total lactate (black bars) and protonated LA (grey bars) in treatment media, the latter calculated using the Henderson-Hasselbalch equation and the treatment media pH (range 6.6–7.1). Values were normalised to account for slight variations in growth rate of bacterial cultures

To evaluate whether LA produced by vaginal *Lactobacillus* spp. could similarly increase expression of tight junction proteins in the context of a complex milieu of bacterial components, VK2 epithelial cells were cultured with media containing 20% (v/v) filtered supernatant from *L. crispatus*, *L. jensenii*, *L. iners* or *G. vaginalis* cultures and gene expression analysed by RNA-Seq. Expression analysis of key tight junction genes described in Fig. 4C revealed a substantial portion of the 54 tight junction genes in this list which were also detected within the bacterial culture-treated cells were differentially expressed following treatment of vaginal epithelial cells with culture supernatants from *L. crispatus* and *L. jensenii* cultures (59% and 56%, respectively, Fig. 7A and Supplementary Table 6). These DEGs included *CLDN4*, *OCLN*, *TJP2* and *F11R* genes shown to be most substantially altered following LA treatment (Fig. 7B). In contrast, less substantial changes to tight junction gene expression were observed following treatment with *L. iners* culture supernatants, with only 19% of genes significantly differentially expressed following treatment and no change to *TJP2* and *F11R* gene expression (Fig. 7 and Supplementary Table 6). Minimal changes to tight junction gene expression were observed following treatment with *G. vaginalis* cultures. Whilst the growth of *L. iners* cultures (4.2 × 10^8^ CFU/mL after 48 h culture) was slightly less than that of *L. crispatus* and *L. jensenii* (5.2 and 5.8 × 10^8^ CFU/ml, respectively), adjusting RNASeq data for these small differences did not materially alter the results (analysis not shown). Media containing 20% *L. crispatus* and *L. jensenii* culture supernatants contained substantially higher levels of both total lactate and protonated LA as compared to that derived from *L. iners* or *G. vaginalis* cultures, even after adjusting for bacterial growth differences (Fig. 7C). These data indicate that supernatants from *Lactobacillus* spp. which produce high levels of LA upregulate the expression of tight junction genes in cervicovaginal epithelial cells in vitro, supporting a specific role for LA in enhancing epithelial barrier integrity.

## Discussion

This study reveals a novel mechanism by which cervicovaginal *Lactobacillus* spp. enhance FRT epithelial barrier integrity through the action of the metabolite LA that directly increases epithelial barrier integrity and expression of tight junction proteins in vitro. The physiological relevance of these findings was indicated in a cohort study which demonstrated an association between high bacterial LDH levels and increased relative abundance of epithelial barrier proteins in young women. Notably, this association was observed after adjusting for several confounders including BV status, nonoptimal bacterial protease relative abundance and *Lactobacillus* spp. abundance, indicating a direct association between L-LDH, which produces lactic acid, and barrier function in vivo. Together, these data suggest LA produced by vaginal lactobacilli may play an important role in enhancing the integrity of the cervicovaginal epithelial barrier in vivo to protect against infection with HIV and other STIs.

Experiments utilising human cervical explant tissue indicate HIV can penetrate both the single-layer columnar epithelial cells of the endocervix and upper FRT as well as the squamous, multilayered ectocervical epithelium of the ectocervix and vagina [20, 62], although studies in macaques indicate preferential virus transduction through the ectocervix and vagina [63]. Cervicovaginal mucus, particularly that produced by women with an *L. crispatus*-dominated microbiota, is able to effectively trap HIV virions [64], and this may represent an important barrier to HIV invasion in regions such as the columnar epithelium of the endocervix [20]. In the squamous epithelium of the ectocervix and vagina, HIV can easily penetrate the superficial epithelial layers of the stratum corneum where intercellular junctional molecules are not intact. However, penetration of HIV beyond the lower layers of epithelial cells expressing intact tight and adherens junction proteins is highly restricted [20, 65]. This suggests the integrity of mucosal epithelial junctions plays an important role in preventing HIV transmission. The data presented here demonstrate a direct LA-mediated enhancement of tight junction protein expression and increased epithelial barrier integrity in an ectocervical epithelial cell line, which may be relevant to HIV infection risk in vivo.

Enhanced barrier integrity of cervicovaginal epithelial cells cocultured with probiotic lactobacilli or conditioned media from *Lactobacillus* spp. cultures in vitro has been demonstrated previously [31, 66]. However, here we show that culture supernatants from *Lactobacillus* spp. producing high levels of LA increased tight junction gene expression in cervicovaginal epithelial cells to a much greater extent than *L. iners*, which produced substantially lower levels of LA. This suggests LA may be a major mediator of these barrier-enhancing effects. Conditioned media from bacterial cultures contain many soluble and insoluble components that may modulate epithelial barriers. Therefore, we used a reductionist approach to examine the specific activities of LA, added directly to cell culture media, on cervicovaginal cells. These studies demonstrate that it is the protonated form of LA, which predominates at pH < 3.86 present in the FRT of women with optimal *Lactobacillus*-dominated microbiota, which possesses epithelial barrier-promoting activity. These effects of protonated LA are uniquely relevant to the FRT as LA produced by intestinal lactobacilli in the gut would be present mostly as lactate due to the near neutral pH of the gut [67]. Whilst it is possible other bacterial-derived products may also contribute to the barrier-promoting properties of *Lactobacillus* spp. culture supernatants, our data suggest LA may be a major bioactive component of this effect.

Here, we observed a similar effect of L- and D-isomers of LA on enhancing epithelial barrier integrity and junctional molecule expression, consistent with our previous findings of comparable immunomodulatory and virucidal properties of both isomers [38, 41]. Indeed, our RNA-Seq analysis indicated that L- and D-LA elicited overlapping transcriptomic changes to ectocervical epithelial cells. *L. crispatus*, which produces predominantly D-LA, has been suggested to be the more favourable commensal *Lactobacillus* spp. due to its greater apparent stability and association with lower levels of genital inflammation and HIV acquisition [10]. D-LA also elicits more potent inhibition of *Chlamydia trachomatis* infection of FRT epithelial cells compared to L-LA in vitro [29]. However, L-LA has been shown to be more potent than D-LA in inactivating HIV at threshold concentrations in vitro [38]. Therefore, whilst our in vitro analysis did not identify differential effects of L- and D-LA on epithelial barrier integrity it remains possible LA isomers may have context and pathogen-specific differential activity in vivo. It is important to also consider that the total concentration of LA (both L- and D-LA) produced by lactobacilli can differ between species [34], which in turn influences pH and thus levels of the biologically relevant protonated form of LA [39]. Indeed, our data indicating *L. crispatus* and *L. jensenii* cultures produced substantially more LA than *L. iners* in vitro was consistent with their ability to upregulate tight junction gene expression to a greater extent.

The in vitro analyses presented in this study demonstrate an epithelial barrier-enhancing effect of LA, whilst our cohort study findings support the in vivo relevance of these observations in the complex FRT environment. Our data indicate that women with a greater abundance of bacterial LDH proteins (measured in ex vivo cervicovaginal samples) have increased expression of proteins important for maintaining epithelial barrier function and tight junction integrity, suggesting that these are physiologically relevant observations. Additionally, the finding that non-*Lactobacillus* taxa were also producing LDH, and that the relationship between LDH and epithelial barrier proteins was independent of BV status, *Lactobacillus* spp. relative abundance and protease/peptidase production by nonoptimal bacteria, suggests a direct relationship between LDH and barrier function. This suggests that although BV-associated bacteria may produce proteases and elicit host inflammatory responses that influence epithelial barrier integrity, the relationship between LDH and barrier function was independent of these factors. Our findings from a larger cohort (*n* = 113) of young South African women at increased risk of HIV support and extend previous observations [46] in Kenyan (*n* = 10) and North American (n = 31) women. Zevin et al. demonstrated enrichment of proteins associated with epidermal development and barrier function in women with *Lactobacillus*-dominated compared to *G. vaginalis*-dominated microbiota as well as a significant positive association between L-LDH abundance and INVO and SPR1A, which are known to act as scaffolding for epidermal layers [46]. Taken together, these in vitro and cohort data suggest a mechanistic link between bacterial-derived LA and cervicovaginal epithelial barrier integrity.

In this study, LA concentrations were inferred from in vivo levels of LDH protein in the metaproteome of women, which enabled the discrimination between relative abundance of host and bacterial LDH. Although the contribution of host versus bacterial LDH to LA measurements is difficult to differentiate, we have previously shown LDH relative abundance correlates directly with levels of total lactate production by lactobacilli in vitro [59]. Furthermore, high bacterial LDH abundance was associated with low vaginal pH. This is in contrast with host LDH, which showed a weak positive association with vaginal pH, consistent with previous findings [68] and may suggest higher levels of cell death (and hence host LDH release) at higher vaginal pH. Furthermore, vaginal bacteria, and not host cells, are considered the primary source of vaginal LA in vivo [43, 69]. Accurate measurement of cervicovaginal LA levels in clinical studies is challenging due to the fact that exposure of cervicovaginal fluid samples to the aerobic environment during collection and processing results in loss of CO_2_ and a subsequent increase in pH that may underestimate the in vivo concentration of the biologically relevant, protonated form of LA [36]. The low prevalence of *L. crispatus* and other D-LDH-producing bacteria in the cohort included in this study may have limited the identification of proteins associated with D-LDH levels. We performed proteomic analysis of cervicovaginal fluid, and although there is a 63% overlap between the FRT tissue and mucus proteome [70], not all cellular junctional molecules are detected using this approach. The majority of the in vitro mechanistic data presented here were performed in an immortalised ectocervical epithelial cell line, and whilst heightened expression of *CLDN1* and *CLDN4* genes was also observed in primary cervicovaginal epithelial cells following LA treatment (Supplementary Fig. 3), confirmation of other results in primary cells, or ideally primary multilayer 3D epivaginal tissue models, is warranted.

Our findings support the potential for LA to be used therapeutically to enhance the natural defences of the FRT mucosa and protect against pathogen infection. The combined abilities of LA to protect against pathogen-mediated inflammation, enhance the epithelial barrier and kill both BV-associated bacterial and HIV and other viruses [71] suggest it may be an ideal agent to promote FRT health. Although a plethora of over-the-counter LA-containing formulations are available for use in the FRT, the formulations and LA content of these products vary considerably, and data on their ability to alter sexual and reproductive health outcomes is limited [72]. However, a recent clinical trial of LA-containing gels in women with recurrent BV demonstrated BV resolution in 47% of participants [73]. Whilst this effect was inferior to that mediated by the standard of care treatment metronidazole alone [74] (70% resolution), LA-containing gels were preferred by women, and side effects were less common [73, 75]. The ability of the probiotic *L. crispatus* CTV-05 to modulate FRT health was recently evaluated in a cohort of women with BV where it was found to significantly reduce the rate of BV-recurrence [76]. Thus, the potential for LA to mediate beneficial in vivo effects is an area warranting future research.

## Conclusions

By utilising a combination of in vitro mechanistic approaches and ex vivo analyses of FRT samples collected from a clinical cohort of young women, this study has identified a novel effect of the *Lactobacillus* metabolite LA on enhancing the integrity of the epithelial barrier through directly increasing levels of junctional molecules. These findings provide important mechanistic insights into the association of *Lactobacillus*-dominated microbiota with favourable sexual health outcomes and indicate potential avenues for therapeutic modulation of the FRT environment to enhance genital health.

## Supplementary Information

**Additional file 1: Figure S1.** Association between cervicovaginal pH and relative abundance of bacterial L- and D-lactate dehydrogenase [LDH; Log2 intensity based absolute quantification (iBAQ) values]. **Figure S2.** Multidimensional scaling (MDS) plot of genes differentially expressed by ectocervical epithelial cells treated with 0.3% L-lactic acid (L-LA) pH 3.9 or D-lactic acid (D-LA) pH 3.9 (yellow and blue respectively), HCl pH 3.9 (green) or untreated cells (red). **Figure S3.** Primary cervicovaginal epithelial cells were apically treated with 0.3% L-lactic acid pH (L-LA, pH 3.9), or low pH media alone (HCl, pH 3.9) for 1 h and gene expression of tight junction factors claudin-1 (CLDN1) and claudin-4 (CLDN4) were assessed 4 h post-treatment by qRT-PCR.

**Additional file 2: Supplementary Table 1.** Proteins with significantly different abundance in women with high as compared to low L-LDH and D-LDH abundance*. **Supplementary Table 2.** Barrier-related proteins with significantly different abundance in women with high as compared to low L-LDH abundance*. **Supplementary Table 3.** Genes differentially expressed by L-LA, D-LA and HCL treatment of Ect cells*. **Supplementary Table 4.** Gene ontology pathways significantly enriched by L-LA and D-LA treatment of Ect cells. **Supplementary Table 5.** Tight junction genes differentially expressed by L-LA, D-LA and HCL treatment of Ect cells*. **Supplementary Table 6.** Tight junction genes differentially expressed by treatment of VK2 cells with bacterial culture supernatants*.

## Data Availability

All data generated during the current study are included in this article (and its supplementary information files). Raw sequence reads will be made available upon request. Metaproteomics data is derived from a previously published cohort study (Alisoltani et al. 2020 Microbiome 8:165) with data available online http://fgtdb.org/. The sequences generated and analysed during this current study were uploaded to the NCBI Sequence Read Archive (SRA) data repository, with project numbers PRJNA823848 and PRJNA835889.
