# Supplementary material for: Lactic acid from vaginal microbiota enhances cervicovaginal epithelial barrier integrity by promoting tight junction protein expression
Source: Microbiome. 2022 Aug 31;10:141. doi: 10.1186/s40168-022-01337-5 (PMC9429363; doi:10.1186/s40168-022-01337-5)
Supplement: Supplementary file 2 — Additional file 1: Figure S1. Association between cervicovaginal pH and relative abundance of bacterial L- and D-lactate dehydrogenase [LDH; Log2 intensity based absolute quantification (iBAQ) values]. Figure S2. Multidimensional scaling (MDS) plot of genes differentially expressed by ectocervical epithelial cells treated with 0.3% L-lactic acid (L-LA) pH 3.9 or D-lactic acid (D-LA) pH 3.9 (yellow and blue respectively), HCl pH 3.9 (green) or untreated cells (red). Figure S3. Primary cervicovaginal epithelial cells were apically treated with 0.3% L-lactic acid pH (L-LA, pH 3.9), or low pH media alone (HCl, pH 3.9) for 1 h and gene expression of tight junction factors claudin-1 (CLDN1) and claudin-4 (CLDN4) were assessed 4 h post-treatment by qRT-PCR. [file 40168_2022_1337_MOESM1_ESM.pdf]

Supplementary Figure 1

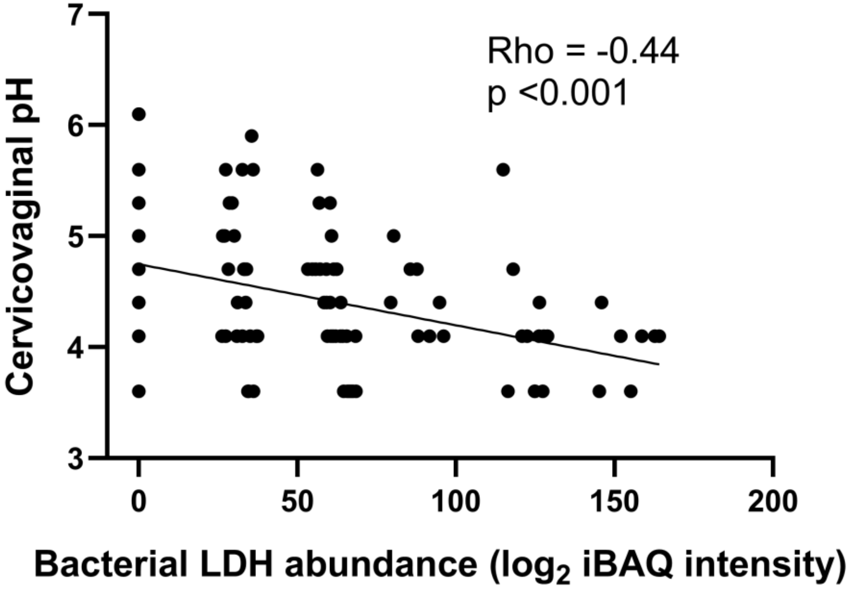

Figure S1. Association between cervicovaginal pH and relative abundance of bacterial L- and D-lactate dehydrogenase [LDH; Log2 intensity based absolute quantification (iBAQ) values]. pH was measured in neat cervicovaginal secretions collected by menstrual cup using pH strips (range: 3.6-8.2). Bacterial LDH relative abundance in lateral vaginal wall swab samples was assessed using liquid chromatography-tandem mass spectrometry. A total of 103 HIV-uninfected non-pregnant women who had both pH and metaproteomics data available were included. Significance assessed by Spearman's rho and $p<0.05$ was considered statistically significant.

Supplementary Figure 2

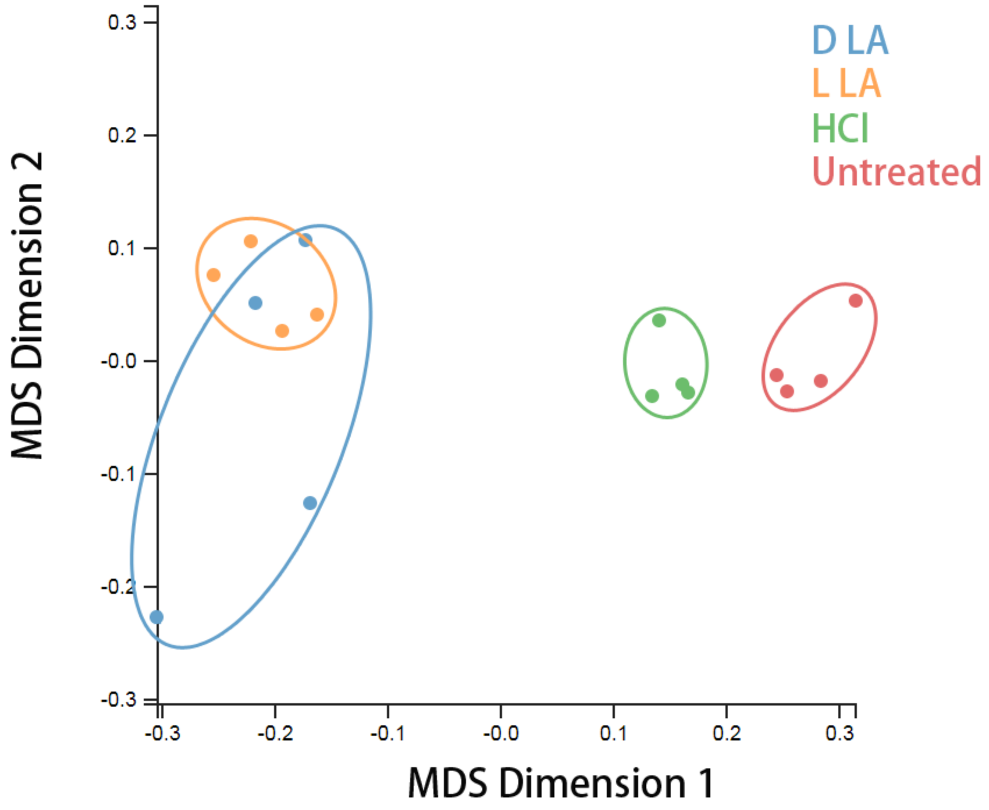

Differentially expressed genes (FDR < 0.01)

Figure S2. Multidimensional scaling (MDS) plot of genes differentially expressed by ectocervical epithelial cells treated with 0.3% L-lactic acid (L-LA) pH 3.9 or D-lactic acid (D-LA) pH 3.9 (yellow and blue respectively), HCl pH 3.9 (green) or untreated cells (red). Graph shows results from 4 independent experiments. False discovery rate ≤ 0.01.

Supplementary Figure 3

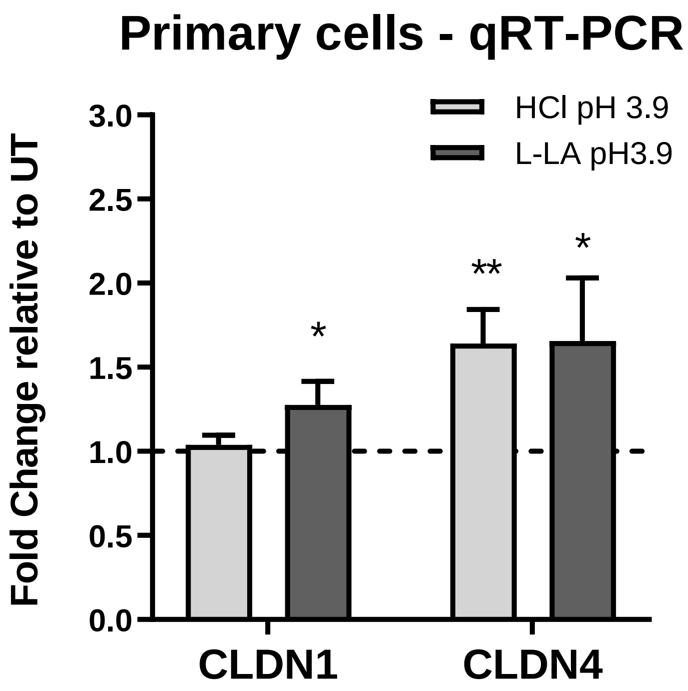

Figure S3. Primary cervicovaginal epithelial cells were apically treated with 0.3% L-lactic acid pH (L-LA, pH 3.9), or low pH media alone (HCl, pH 3.9) for 1 h and gene expression of tight junction factors claudin-1 (CLDN1) and claudin-4 (CLDN4) were assessed 4 h post-treatment by qRT-PCR. Graphs indicate relative gene expression (fold change) as compared to untreated cells; mean +/- SEM from n=4 independent experiments. * and ** represent $p<0.05$ and <0.01 respectively as compared to untreated cells as determined by Mann-Whitney U test.
